# Investigating the impacts of COVID-19 among LGBTQ2S youth experiencing homelessness

**DOI:** 10.1371/journal.pone.0257693

**Published:** 2021-09-21

**Authors:** Alex Abramovich, Nelson Pang, Amanda Moss, Carmen H. Logie, Michael Chaiton, Sean A. Kidd, Hayley A. Hamilton

**Affiliations:** 1 Institute for Mental Health Policy Research, Centre for Addiction and Mental Health, Toronto, Ontario, Canada; 2 Dalla Lana School of Public Health, University of Toronto, Toronto, Ontario, Canada; 3 Factor Inwentash Faculty of Social Work, University of Toronto, Toronto, Ontario, Canada; 4 Women’s College Research Institute, Women’s College Hospital, Toronto, Ontario, Canada; 5 Department of Psychiatry, University of Toronto, Toronto, Canada; University of Perugia: Universita degli Studi di Perugia, ITALY

## Abstract

**Background:**

LGBTQ2S youth are overrepresented among youth experiencing homelessness and experience significantly higher rates of mental health issues compared to heterosexual and cisgender youth. COVID-19 related challenges for LGBTQ2S youth experiencing homelessness remain unknown. To address this gap, this study aimed to understand the impacts of the COVID-19 pandemic on LGBTQ2S youth at risk of, and experiencing, homelessness in the Greater Toronto Area, Ontario, Canada and surrounding areas.

**Methods:**

Utilizing a mixed-methods convergent parallel design, LGBTQ2S youth experiencing homelessness were recruited to participate in virtual surveys and in-depth one-on-one interviews. Surveys included standardized measures and were administered to measure mental health outcomes and collect information on demographic characteristics, and health service use. Survey data were analyzed with descriptive statistics and statistical tests for difference of proportions. Interviews were analyzed using an iterative thematic content approach.

**Results:**

Sixty-one youth completed surveys and 20 youth participated in one-on-one interviews. Quantitative and qualitative data showed that youth have been significantly impacted by the COVID-19 pandemic in various ways, including experiencing poor mental health, such as suicidality, depression, anxiety, and increased substance use, and lack of access to health and social support services.

**Conclusion:**

Our study highlights the need for LGBTQ2S inclusive and affirming health care and support services for precariously housed adolescents to address the pre-existing social and health issues that have been exacerbated by the pandemic.

## Introduction

The COVID-19 pandemic has had damaging effects on the mental health of young people, who are more likely than others to face symptoms of depression and anxiety during this time [1–3]. Youth-serving organizations in high income contexts such as Canada are currently facing numerous challenges meeting the needs of youth, due to a decreased availability of staff and programs. Services typically available to youth in crisis have had to close their doors and are no longer accepting new clients, making it particularly difficult for youth to access new services or maintain the support they rely on [4]. For instance, a recent report in Toronto, Canada focused on the youth-serving sector found that unhoused and provisionally accommodated youth are experiencing ongoing challenges meeting their basic needs, including food and shelter, during the COVID-19 pandemic [4]. These issues are likely exacerbated for vulnerable subpopulations of youth, who may have struggled with mental health challenges and barriers accessing services prior to the COVID-19 pandemic, such as lesbian, gay, bisexual, transgender, queer, questioning, and 2-spirit (LGBTQ2S) youth.

LGBTQ2S youth are overrepresented among youth experiencing homelessness and constitute 20–40% of the homeless youth population in North America [5–9]. LGBTQ2S youth experience significantly higher rates of mental health issues compared to heterosexual and cisgender youth across global contexts, due to stigma, discrimination, and identity-based rejection [5, 7, 10, 11]. Public health measures, such as physical distancing and the closure of social support programs, have undoubtedly resulted in negative outcomes among LGBTQ2S youth at risk of, and experiencing, homelessness. Unique stressors faced by LGBTQ2S youth during the COVID-19 pandemic include being forced to isolate at home with unsupportive and abusive family members, due to a lack of alternative housing options, which can have devastating effects on these young people [12, 13]. A recent U.S. study reported reduced access to in-person services and increased access to virtual population-based crisis services among LGBTQ2S youth; however, there was a reluctance to engage in telehealth-based services, due to fear of parents or family members overhearing their conversations [14]. Loss of access to safe, supportive, and affirming environments and relationships are cause for concern, given that they have been found to be key protective factors for suicide, self-harm, and depression among LGBTQ2S youth [10, 14, 15].

Adverse effects of the pandemic on mental health, food security, substance use, and employment have been observed among LGBTQ2S individuals in Canada and the U.S. [16, 17]. However, COVID-19 related challenges for LGBTQ2S youth experiencing homelessness remain unknown. To address these gaps, this ongoing study engaged a group of LGBTQ2S youth at risk of, or experiencing, homelessness in the Greater Toronto Area (GTA) and surrounding areas, Canada to understand their specific challenges, coping strategies, and mental health responses during the COVID-19 pandemic.

## Methods

This mixed-methods study utilized a convergent parallel design [18]. This design was selected because it is effective in ensuring required data is collected within a limited timeframe to understand needs of specific populations during a public health emergency and to develop recommendations for local policy makers and service providers [19]. This study is part of a larger longitudinal study that aims to understand the impacts of the COVID-19 pandemic on LGBTQ2S youth at risk of, and experiencing, homelessness in the GTA and surrounding areas.

### Participants

A rolling enrollment strategy was used to recruit 61 LGBTQ2S youth who were either at risk of, or experiencing, homelessness to participate in this study. Criteria for inclusion were: self-identify as LGBTQ2S; aged 14–29; at risk of, or experiencing, homelessness (living with unsupportive family; struggling to pay rent; staying at a shelter or housing program, etc.); living in the GTA or surrounding areas (Toronto, Durham, Halton, Peel, York Region, Hamilton, Barrie, Guelph, Kitchener, Waterloo, Cambridge). Homelessness describes a range of housing and shelter experiences including being unsheltered, emergency sheltered, provisionally accommodated, and being at-risk of homelessness. A young person is considered to be experiencing homelessness when they are living independently of parents/caregivers, but are unable to secure stable, safe, or consistent housing [20]. Similarly, a young person is considered to be at-risk of homelessness when their housing situation is dangerously lacking security or stability [21]. This study includes youth up to the age of 29, in line with the Government of Canada’s definition of youth (up to the age of 29) [5].

### Quantitative methods

#### Sampling

Using a convenience sampling method, we recruited participants by collaborating with youth serving organizations and hiring a Peer Support Worker to coordinate online recruitment through virtual information sessions and paid social media advertisements. The Peer Support Worker identified as LGBTQ2S and was in the same age range as the participants. They were mainly responsible for recruitment and also contributed to the creation of data collection materials. Youth were asked to contact the study team if interested in participating. A $35 electronic gift card was provided as honoraria to each participant upon completion of the baseline survey. Findings based on data collected from all youth participants (n = 61) enrolled in the study up to mid-April 2021 are included in this article.

#### Data collection

Upon completion of a rigorous screening process to ensure participants met inclusion criteria, unique survey links were sent to participants. Written informed consent was obtained from each participant prior to the beginning of the survey. The lack of parental or guardian consent was approved by the Research Ethics Board. Sixty-one youth consented and enrolled in the study and completed the first (baseline) of three virtual surveys. The survey contained questions on demographic characteristics, impacts of the COVID-19 pandemic, health service use, and health outcomes, including depression, anxiety and suicidality, and took approximately 30 minutes to complete. Validated and standardized measures were used to measure mental health outcomes, including the General Anxiety Disorder-7 item scale (GAD-7) for anxiety, the Patient Health Questionnaire (PHQ-9) for depression, and the CAGE-AID Questionnaire adapted to screen for alcohol and drug use [22–26]. For the GAD-7, cutoff scores of 5, 10, and 15 represented mild, moderate, and severe anxiety, respectively [23]. For the PHQ-9, cutoff scores of 5, 10, 15, and 20 represented mild, moderate, moderately severe, and severe depression [24]. For the CAGE-AID screening tool, a score of two or more indicated problematic alcohol and/or substance use [25]. Suicidality was measured using a scale derived from a four-item scale previously used in studies with youth. [27, 28] Each item (e.g., how often in the past year participants had thought about killing themselves, death/dying) was answered on a four-point scale with responses ranging from never to all the time.

Data collection commenced in January 2021, during the second wave of COVID-19, at a time where lockdown restrictions were in place. Throughout the pandemic, lockdown restrictions across Canada have varied by province. At the beginning of data collection in January 2021, the Government of Ontario declared a second provincial emergency, which included: a stay-at-home order; requirement to wear a mask or face covering in the indoor areas of businesses or organizations that are open and outdoors when unable to physically distance more than two metres; reduction of outdoor gatherings to a limit of 5 people; reduction of hours for non-essential businesses; and students required to attend classes online. The provincial emergency expired in February 2021, but the stay-at-home order was extended. Lockdown restrictions began to ease in March 2021 in lower COVID-19 case count regions in Ontario (York Region, Durham, Hamilton) including the opening of some non-essential businesses and schools. However, in April 2021, the Government of Ontario issued a province-wide shutdown resulting in closures of in-person classes at school and non-essential businesses, and residents were recommended to remain home unless performing essential activities. Ontario’s stay-at-home order expired in June 2021 and the province entered Step 1 of its re-opening plan. Survey data was collected and managed using REDCap electronic data software hosted at the Centre for Addiction and Mental Health (CAMH) [29, 30].

#### Analysis

All analyses were conducted in R version 3.6.3 [31]. Data was analyzed using descriptive statistics and statistical tests for difference of proportions. Due to small sample sizes, we were unable to test for differences between gender identity, sexual orientation, and ethno-racial identity groups.

### Qualitative methods

#### Sampling

Upon completion of the baseline survey, youth were invited to participate in virtual one-on-one interviews. A $40 electronic gift card was provided as honoraria to each participant upon completion.

#### Data collection

Twenty youth participated in virtual, semi-structured, in-depth, one-on-one interviews (approximately 60 minutes) using a secure video conferencing platform. Virtual interviews were conducted by one Research Analyst, with a Master of Social Work degree. Written informed consent was obtained from each participant again prior to the beginning of their interview, whereby the Research Analyst read aloud the consent form and answered any questions that were raised. The interview guide included questions focused on COVID-19 related challenges and barriers, mental health, coping strategies, family life, and access to services (S1 File). Interviews were audio-recorded and transcribed verbatim.

#### Analysis

Qualitative data analysis occurred in conjunction with data collection to investigate emergent themes and the interview guide was amended as necessary. Qualitative data was coded and analyzed by two research team members using an iterative thematic content approach [32, 33]. Data analysis involved identifying core themes, data patterns, and developing codes that helped explain the themes. Any information that could personally identify respondents was removed from the interview transcripts and replaced with pseudonyms, which are used throughout this article to conceal the identity of participants. The preliminary analysis involved open coding to generate a range of key themes that emerged from the data. These initial codes were then organized into provisional categories to build a coding frame divided into major themes and sub-themes.

### Mixed-methods data integration

Following a convergent parallel analytical design, qualitative and quantitative data were analyzed separately, then merged for interpretation [34, 35]. The data sources and methodologies were triangulated to confirm, cross-validate, and corroborate findings within and between participants [33]. The integrated interpretation of the quantitative and qualitative results is presented in the following section. Qualitative results are reported according to COREQ guidelines [36].

### Ethics

Ethics approval was obtained from the CAMH Research Ethics Board (#102/2020).

## Results

The sociodemographic characteristics of the study sample are reported in Table 1. Participants had an average age of 21 years and represented diverse ethno-racial backgrounds including Indigenous, Black, Asian, mixed-background, and White. The majority of participants identified their gender as transgender or gender diverse, and sexual orientation as bisexual. Youth also described their sexual orientation as gay, lesbian, pansexual, queer, and asexual. The average age at which participants first experienced homelessness was approximately 16 years.

**Table 1:** Socio-demographic characteristics of sample (n = 61).

Age Category	n (%)
16–20	34 (55.74%)
21–24	16 (36.23%)
25–29	11 (18.03%)
**Gender Identity**	
Cisgender Woman	20 (32.79%)
Cisgender Man	5 (8.20%)
Transgender Woman	7 (11.48%)
Transgender Man	11 (18.03%)
Gender Diverse (two-spirit, non-binary, genderfluid)	15 (24.59%)
Missing	<5
**Sexual Orientation**	
Asexual	<5
Bisexual	22 (36.07%)
Gay	12 (19.67%)
Lesbian	9 (14.75%)
Pansexual	6 (9.94%)
Queer	8 (13.11%)
Straight/Heterosexual	<5
Missing	<5
**Ethno-Racial Background** (Select all that apply)	
Indigenous	<5
Black/African/Caribbean	11
Asian (East, South, West)	6
Latinx	<5
White/European	40
Mixed-Background	7
**Employment** (Which of these best describes your current work situation? Select all that apply.)	
Employed	22
Unpaid work	8
Unemployed	34
Long-term sick or disabled	6
Student	18
**Main source of income since start of pandemic** (after March 1, 2020)	
Ontario Works	19 (31.15%)
Ontario Disability Support Program	7 (11.48%)
Self-employed/freelance work	<5
Part-time employment	6 (9.84%)
Full-time employment	<5
CERB/EI/CESB	9 (14.75%)
Not listed	13 (21.31%)
**Housing** (Select all that apply since COVID-19)	
A place you rent	16
Parents’/caregivers’ place	20
Friends’ or partners’ place	35
Emergency/domestic violence shelter	14
Supervised residence/transitional housing	17
Public space (vehicle, makeshift shelter, vacant building)	20
Motel or hotel	<5

**Fig 1:**
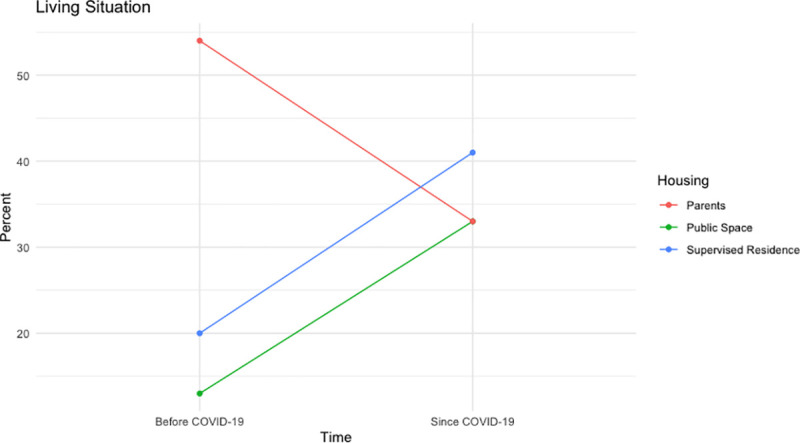
Changes to living situation since the beginning of COVID-19.

The vast majority of participants reported changes to their housing situation since the COVID-19 pandemic began (see Fig 1). For example, some participants spoke about couch surfing at the homes of friends’ parents prior to the pandemic, which was no longer an option, due to public health measures. A common narrative among participants was that they experienced significant difficulties securing safe, appropriate and affordable housing throughout the pandemic. Stevie, 16-years-old, spoke about their struggles finding housing during the pandemic:

*“I had a rough time finding places to go when the pandemic happened, a lot of opportunities and just resources shut down for me. I can definitely say before the pandemic I had a lot more options than I did when it happened.”*

At the time of the baseline survey, youth reported living temporarily with parents, emergency shelters, transitional housing programs, couch surfing, and in public spaces. Approximately 20% of participants were staying at a shelter, transitional housing program or group home prior to the pandemic, compared to ~41% of youth since the pandemic (p<0.01). Prior to the pandemic, ~13% of youth reported living in a public space, vehicle, or vacant building, compared to ~33% of youth since the pandemic began (p<0.05). One participant reflected on the state of encampments in Toronto since the COVID-19 pandemic:

*“Before I never noticed all these tents and encampments in public parks [*…*] I heard from a friend*, *even in the graveyard*, *they’re pitching tents in the graveyard and they’re living there*. *So*, *like*, *it’s really getting out of control*.*”* (Jerry, 24-years-old)

Over half of the youth participants (~54%) were living with their parents pre-pandemic compared to ~33% of youth who reported living with their parents since the pandemic (p<0.05). CeCe, 26-years-old, reflects on their experience of being isolated with unsupportive parents:

*“You’re isolating 24/7 with your emotional and psychological and religious abusers and that has been pretty awful to say the least. I’ve had nervous breakdowns, I’ve had panic attacks, I’ve had anxiety attacks, I’ve had very severe depressive episodes […] I already suffered with depression and fatigue even before the pandemic, but the fact that I’m constantly being triggered all the time, almost every single day because I’m surrounded by so many religious triggers, it’s not healthy […] And there is no social escape, there is no physical escape.”*

Numerous youth reported losing their employment, as a result of the COVID-19 pandemic (~28% unemployed pre-pandemic vs ~56% since pandemic started, p<0.01), and were, therefore, unemployed or doing unpaid work. The majority of participants (~57%) reported that their main source of income included Ontario Works (OW), Ontario Disability Support Program (ODSP), Canada Emergency Response Benefit (CERB), Employment Insurance (EI), or Canada Emergency Student Benefit (CESB). Ori, 24-years-old, shared the impact of job loss on their mental health and substance use:

*“I’m a very busy person, I’m a workaholic. I always need to be doing something…So when COVID happened and everything was shut down, I was really, really struggling. I did not know what to do with myself. That’s when, you know, the drinking, drugs, everything started happening.”*

### COVID-19 impact on mental health and substance use

#### Mental health

Participants reported experiencing a multitude of mental health difficulties and challenges they related to the COVID-19 pandemic (See Table 2). The majority of respondents reported experiencing poor mental health, self-harm, and suicidality since the pandemic. Approximately 81% (n = 50) of youth engaged in non-suicidal self-injury and ~36% (n = 22) of participants reported attempting suicide since the start of the COVID-19 pandemic. For example, one participant reflected on the pandemic’s impact of the increased social isolation on their mental health, resulting in a suicide attempt:

*“The beginning of the pandemic and like stretching in for the months after that was extremely difficult for me because the transition from being like with people all the time and constantly being out and having fun and hanging out with my friends to all of a sudden*, *just having nothing*, *that was devastating for me*. *And then when everything started getting cancelled and stuff*, *it was such a blow to my mental health for a very long time*. *I was in the psych ward at one point for just how bad it had gotten for me…I attempted to take my own life*.*”* (Xavier, 16-years-old)

**Table 2:** Impact of COVID-19 on mental health.

Since the start of the COVID-19 pandemic, have you hurt or injured yourself on purpose without wanting to die (i.e. cutting, burning, or bruising yourself on purpose)?	n (%)
Yes	50 (81.97%)
No	11 (18.03%)
**Since the start of the COVID-19 pandemic, have you thought about attempting suicide?**	
Yes	47 (77.05%)
No	13 (21.31%)
Missing	1 (1.64%)
**Did you attempt suicide in the past 12-months?**	
Yes	22 (36.07%)
No	39 (63.93%)
**Is your life lonelier because of the COVID-19 pandemic?**	
Yes	59 (96.72%)
No	1 (1.64%)
Missing	1 (1.64%)

**Fig 2:**
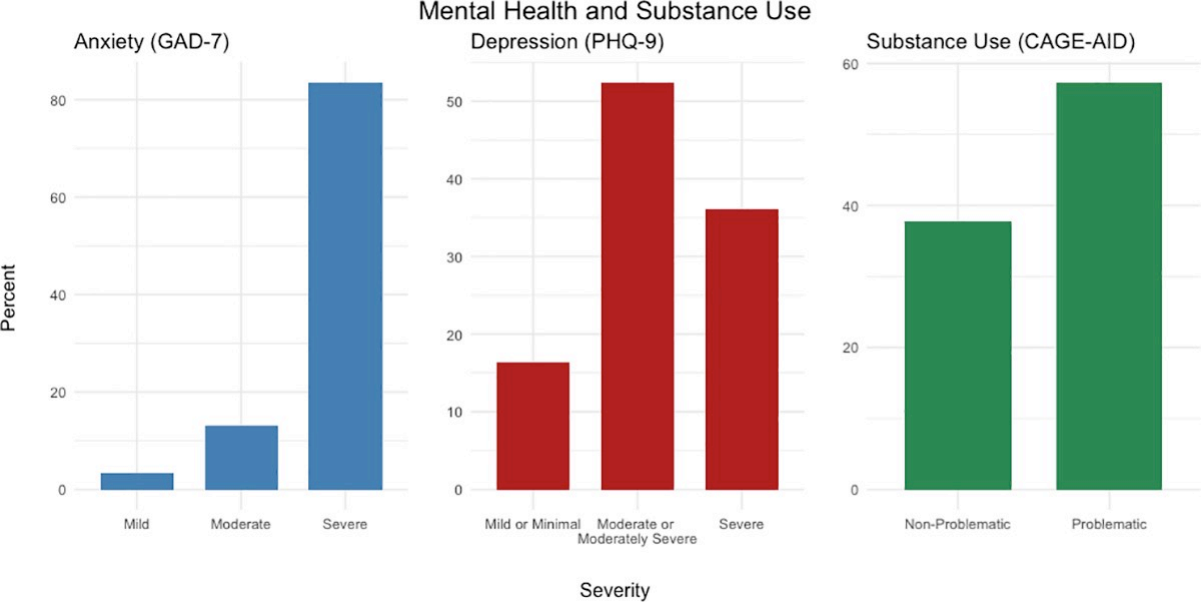
Mental health and substance use among participants.

All respondents (n = 61) reported experiencing anxiety and ~84% (n = 51) scored in the ‘severe anxiety’ range on the GAD-7 anxiety scale [21]. Likewise, all youth reported experiencing symptoms of depression, with ~66% (n = 40) scoring in the ‘moderately severe’ or ‘severe depression’ range on the PHQ-9 depression scale [22] (see Fig 2). Participants described ongoing situations throughout the pandemic that resulted in poor mental health, including staying with unsupportive parents or relatives, overall pandemic stress, and not being able to access safe and affordable housing. Anita, 26-years-old, discussed how their stress and anxiety increased since the pandemic started:

*“My stress levels were already pretty high before the pandemic, but they were manageable in the sense that I didn’t feel like I was on the verge of a panic attack every day, but then beginning of the pandemic, I felt like they had just kind of skyrocketed at some points where I was having nights where I couldn’t sleep because of heart palpitations.”*

In addition to mental health issues, suicidality, and stress, almost all youth (n = 59, ~97%) reported that their lives are lonelier because of the COVID-19 pandemic. Overall, mental health was a major concern perceived by the majority of youth participants.

#### Substance use

**Fig 3:**
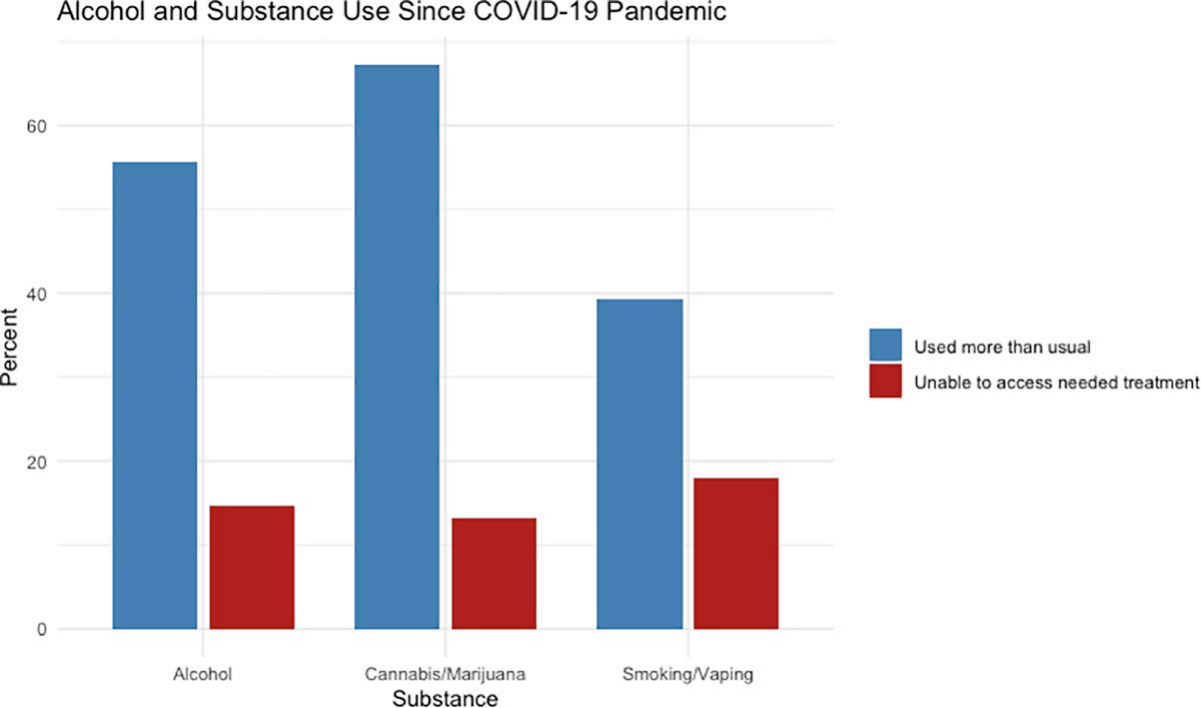
Alcohol and substance use among participants.

The interview and survey data revealed that increased alcohol and substance use, since the beginning of the COVID-19 pandemic, was a predominant issue identified by most youth participants (see Fig 3). Participants described job loss, difficult living situations, loss of connection to friends and community, and identity-based discrimination as factors that contributed to their increased alcohol and substance use. One participant reflected on the devastating impact substance use has had on their community:

*“In my community*, *we’ve been seeing a lot of people overdosing*. *A lot of people are getting more into opioids like fentanyl and there’s this new fentanyl on the streets called ‘carfentanil’*, *which is even worse*. *And I’m finding that a lot of people are overdosing or they’re dying [*…*] They couldn’t get a job*, *they couldn’t interact with people*, *they had no connection [*…*] It’s been really hard because in our community we’ve lost some people*, *you know who were sober for years and unfortunately it was just the pandemic that kind of hit them and they couldn’t cope*.*”* (Lily, 20-years-old)

Problematic alcohol and substance use was measured using the CAGE questionnaire [26]. The majority of youth (n = 35; ~57%) scored in the range of clinically significant for problematic alcohol and/or substance use. Most youth (n = 34, ~56%) reported consuming more alcohol than usual and ~44% (n = 15 out of 34) consumed a lot more alcohol than usual since the pandemic. Approximately 13% (n = 8) of youth reported having had alcohol poisoning since the beginning of the COVID-19 pandemic, while ~28% (n = 17) had concerns about overdosing or having alcohol poisoning in the future. Approximately 39% (n = 24) of youth reported smoking/vaping more than usual and ~46% (n = 11 out of 24) reported smoking/vaping a lot more than usual since the beginning of the pandemic. A high proportion of youth (n = 41; ~67%) reported using more cannabis/marijuana than usual and ~41% (n = 17 out of 41) of youth reported using a lot more cannabis/marijuana than usual since the pandemic. The majority (n = 32; ~52%) of youth relied on cannabis/marijuana more frequently outside of a medical purpose. Jack, 21-years-old, reflected on their increased substance use and dependence:

*“I definitely have increased my substance use. I’ve gotten very into marijuana, like very into marijuana, to the point where it has definitely been one of the biggest things I spend money on and probably the reasons why I don’t have money right now…. I find myself needing to smoke weed to get through my day, which was never a problem before. I like to be very sober, especially when I’m studying, and that has proven to be an issue. So yeah, substance use is a horrible thing and it’s definitely increased.”*

### Impact on health care access

The majority of youth (n = 51, ~84%) experienced changes to their access to medical health care since the start of the COVID-19 pandemic. For example, ~46% (n = 28) of youth reported missing a scheduled appointment with a healthcare provider over the past month for a variety of reasons, including cancelled appointments due to COVID-19, forgetting to go to appointments, and to avoid other people. Three-quarters of youth (n = 45; 74%) reported delaying or not getting the health care they thought they needed. Approximately 33% (n = 20) of youth reported mild changes, such as appointments being moved online instead of in-person visits. A quarter of participants (n = 15; ~25%) had moderate changes, such as delayed appointments or delays getting prescriptions, which impacted their health, while ~16% (n = 10) reported severe changes and were unable to access needed care, resulting in negative impacts to their health. Similarly, the majority (n = 53; ~87%) of youth experienced changes to their access to mental health care since the start of the COVID-19 pandemic. Approximately one-third (38%; n = 23) reported mild changes, such as appointments being moved online instead of in-person and ~13% (n = 8) had moderate changes, such as delayed appointments or delays getting prescriptions, which impacted their mental health. Whereas ~31% (n = 19) reported severe changes and were unable to access the care they needed, resulting in negative impacts to their mental health.

Numerous youth (n = 24; 39%) had to postpone a medical procedure due to the COVID-19 pandemic. Among transgender participants, 64% reported having to postpone or cancel transition-related medical appointments (n = 14 out of 22) and 50% (n = 11 of 22) had to postpone or cancel a transition-related surgery. Postponed and/or cancelled transition-related medical care were described as having detrimental impacts on the health of transgender youth, such as depression, anxiety, and suicidality. For example, one participant discussed the experience of having their transition-related surgery cancelled:

*“I was supposed to hear back with a surgery date by the end of March for bottom surgery*, *and then towards the end of March*, *I got the email that the place in Montreal basically closed*. *So*, *I wasn’t able to have surgery until September and the March thing was something I was looking forward to*, *counting down the days and then everything just came to a screeching halt*. *The day I found out I was relatively suicidal*, *like I’m just going to opt myself*.*”* (Luna, 23-years-old)

Participants described numerous barriers accessing counselling and/or support groups since the start of the pandemic, resulting in ~62% (n = 38) of youth being unable to access counselling and/or support groups. Similarly, youth reported being unable to access alcohol and substance use treatment. Since the beginning of the pandemic, approximately 18% (n = 11) of youth were unable to get the help they needed to stop smoking, ~15% (n = 9) of youth were unable to access alcohol treatment, and ~13% (n = 8) were unable to access help to stop using cannabis/marijuana.

### Virtual care

The COVID-19 pandemic has resulted in numerous health care services being offered online. Two-thirds (n = 40; 66%) of youth reported accessing mental health services virtually, nearly half (n = 30; ~49%) of youth accessed case management virtually, ~43% (n = 26) of youth accessed crisis services virtually, and ~34% (n = 21) of youth accessed legal services virtually. The shift to virtual care was perceived as having benefits and disadvantages for youth participants.

Among the benefits of virtual care, youth reported saving money on transportation costs and decreased levels of social anxiety. Several youth described feeling less vulnerable to judgement. For example, one participant spoke about their comfort levels increasing with virtual care and worrying less about being judged about his gender identity.

*“As a trans guy*, *I sometimes feel like it’s more comfortable to do it virtually because I skipped the part that people can judge me*. *For example*, *do you have any idea how tall I am right now*? *No*, *you don’t*. *And like*, *it’s kinda like* [the] *same measure for everyone else because you can adjust your camera and everyone looks kinda like this*, *like the size doesn’t matter anymore*.*”* (Edi, 20-years-old)

CeCe, 26-years-old, shared how online services can increase accessibility:

*“I avoid transportation expenses. I avoid having to get dressed up. I avoid triggering my social anxiety because they don’t have to see me. I don’t have to see them. So, there are some pros to virtual accessibility and the ease of phone accessibility.”*

On the contrary, some youth described logistical barriers, including issues with their internet connection, and others spoke at length about living with unsupportive parents and/or family members, making it difficult, and in some cases, not possible to access virtual care. For example, one participant stated:

*“It’s been very hard for me to find that information by myself*, *you know and especially if I was looking*, *a lot of things do pop up*, *but then there’s the restrictions to the pandemic*, *you know*, *there’s the restrictions to*, *will I have WIFI*? *Will I be able to have those resources to find out the information and if I do find out the information*, *do I have a phone plan to make a call to someone*?*”* (Stevie, 16-years-old)

Jordan, 17-years-old, reflected on some of the challenges they have faced trying to access services due to a lack of privacy, while living with homophobic family members, during the COVID-19 pandemic:

*“I’m in my house and if I’m not in the lowest corner of my basement my family can hear me and I don’t want that. So, the pandemic has made it harder to get help in the first place and then once I have it, it’s harder to talk about it and to figure out things that work.”*

Participants reported varied experiences accessing virtual care. On one hand, virtual care created a more accessible service system for some, but on the other hand, it resulted in major privacy concerns, resulting in inaccessibility issues for others.

## Discussion

In this study we aimed to understand the specific challenges, coping strategies, and mental health responses among LGBTQ2S youth at risk of, or experiencing, homelessness during the COVID-19 pandemic. Our findings suggest that COVID-19 has had a negative impact on various aspects of participants’ lives, including housing, health, and access to services. Based on our results, numerous participants experienced changes to their housing situations since the COVID-19 pandemic began. For example, there was a significant increase in the number of youth living in public spaces, vehicles, and vacant buildings, as well as emergency shelters and housing programs since the pandemic compared to prior to the pandemic. Youth also reported living with unsupportive or abusive parents and/or family members. Numerous youth were unable to secure safe, affirming, and affordable housing during the COVID-19 pandemic, due to the reduced availability of housing options, including supervised residences, such as group homes. LGBTQ2S youth at risk of, and experiencing, homelessness frequently couch surf, as a result of the lack of safe and inclusive housing options; however, the pandemic has eliminated this option for many. Previous research has supported housing as an essential social determinant of health [37]. The lack of safe and inclusive housing options for LGBTQ2S youth experiencing homelessness have serious negative consequences on their physical and mental health [38].

The survey data revealed that almost all youth participants experienced poor mental health, including suicidality, depression and anxiety, and increased substance use during the pandemic. Similar to our findings, previous studies have reported that LGBTQ2S youth experiencing homelessness experience high rates of mental health issues, substance use, and suicidality [5, 39]. Despite the already elevated rates of mental health and substance use concerns prior to COVID-19, our findings suggest that the pandemic has had a significant impact on the mental health and wellbeing among LGBTQ2S youth at risk of, and experiencing, homelessness. Our study also demonstrated an increase in detrimental health outcomes, including self-harm, and problematic alcohol and substance use. This is worrisome considering that most participants reported being unable to access the care they needed.

Transgender youth face significant physical and mental health disparities compared to cisgender youth, including higher rates of suicidality, substance use, depression, and anxiety [40–42]. Most transgender participants in our study reported that their transition-related medical appointments, including access to gender-affirming interventions, such as transition-related surgery and hormone therapy, had been postponed or cancelled since the COVID-19 pandemic began. Previous studies reveal that body dysphoria, depression, and suicidality decrease among transgender youth following engagement in gender-affirming interventions, such as hormone therapy and transition-related surgery [43–46] and that delaying gender-affirming treatment is associated with increased psychiatric comorbidity [47, 48].

Our findings reveal that numerous integral health care, social support, and housing services previously available to LGBTQ2S youth at risk of, and experiencing, homelessness have closed their doors or are not accepting as many clients due to the COVID-19 pandemic. These program and service closures have resulted in most participants being unable to access and receive necessary and appropriate care. Although our results highlight major barriers accessing care during the COVID-19 pandemic, we also found that numerous services have adapted to the pandemic and are offering virtual care. Several youth reported positive experiences accessing services virtually, such as reduced social anxiety and saving on transportation costs. However, there were ongoing challenges and barriers experienced by others, including privacy concerns, not having access to the internet or internet connection issues, and difficulty navigating online services. Privacy concerns were a common challenge and barrier for many youth, particularly those who lived in a shared residence or with unsupportive family members. These findings align with calls for urgent attention to LGBTQ2S wellbeing in the pandemic across global contexts [49].

Our results also suggest that there is an urgent need for preventive and longer-term LGBTQ2S specialized mental health support and treatment, in addition to emergency/crisis services. Participants spoke directly to the need for LGBTQ2S inclusive and affirming services and service providers. Efforts are required to ensure that services are more accessible to youth experiencing homelessness and to those unable to access services virtually.

### Strengths and limitations

To our knowledge, this is the first study to specifically investigate the impacts of COVID-19 on LGBTQ2S youth at risk of, and experiencing, homelessness. A major strength of our study was the mixed-methods convergent parallel design, which included valid and standardized measures to assess mental health outcomes and in-depth one-on-one interviews to understand participants’ experiences and perspectives.

Our study has several limitations. The key limitation is that LGBTQ2S youth at-risk of, and experiencing, homelessness have been found to experience high rates of mental health issues and housing precarity prior to the COVID-19 pandemic, which makes it difficult to attribute some of the results directly to the effects of the pandemic, introducing a sampling bias.

Although we worked with a wide range of organizations and community partners to recruit a diverse and representative sample of LGBTQ2S youth, we experienced challenges with participant recruitment. Recruiting a large and diverse sample of participants was difficult, given the prevalence of hidden homelessness among LGBTQ2S youth. As a result, our sample contains many youth who were connected to services and able to access technology. Youth without access to technology were unable to participate in our study, which may have excluded some of the most marginalized LGBTQ2S youth. Therefore, we are unable to determine the extent to which our quantitative sample reflects the broader population of LGBTQ2S youth at risk of, and experiencing, homelessness during the COVID-19 pandemic. Our sample was also too small to conduct analyses of different experiences by gender identity and sexual orientation, along with other social identities including ethno-racial identity. The cross-sectional study design precludes understanding causality and directly attributing the social and health issues raised by participants to the effects of COVID-19.

## Conclusion

This study found that LGBTQ2S youth at risk of, and experiencing, homelessness have been significantly impacted by the COVID-19 pandemic in various ways, particularly their mental health and access to health and social support services. Our study highlights the need for LGBTQ2S inclusive and affirming health care and support services for precariously housed adolescents to address the pre-existing social and health issues that have been exacerbated by the pandemic.

## Supporting information

S1 File Semi-structured interview guide. (PDF)
