# Supplementary material for: Investigating the impacts of COVID-19 among LGBTQ2S youth experiencing homelessness
Source: PLoS One. 2021 Sep 21;16(9):e0257693. doi: 10.1371/journal.pone.0257693 (PMC8454954; doi:10.1371/journal.pone.0257693)
Supplement: S1 File — (PDF) [file pone.0257693.s001.pdf]

S1 File: Semi-structured Interview Guide

1. Where are you currently living?
2. Were you living there prior to the COVID-19 pandemic (before March 1, 2020)?
3. Did you move at any point during the COVID-19 pandemic, including being moved into a hotel program?
4. What has your experience been like living (insert living situation) during the pandemic? Please describe any challenges that you have faced.
5. Did your gender identity or sexual orientation have anything to do with you becoming street- involved or homeless, or being at-risk of homelessness?
6. Are you currently employed?
7. The next question will be about sex work. Has the COVID-19 pandemic caused you to engage in, or engage more in sex work or survival sex (trading sex for a place to sleep)?
8. Have you received the COVID-19 vaccine?
9. The next questions will focus on family, and particularly, family violence. As a child, were you ever involved with child protection services/Children’s Aid/child welfare system?
10. Do you have contact with your family (e.g., parents, aunt, uncle, grandparents, cousins)?
11. Prior to the COVID-19 pandemic, did you experience any abuse, maltreatment, neglect, or identity-based rejection by members of your family?
12. Has your overall stress changed following the COVID-19 pandemic?
13. How has your mental health changed since COVID-19?
14. Has your alcohol, marijuana, substance, or tobacco use changed since COVID-19?
15. How has your physical health changed since COVID 19?
16. What has been most difficult to deal with about the COVID-19 pandemic?
17. How do you feel about mandatory masks and physical distancing measures put in place?
18. Has it been difficult to comply with public health measures (masks, distancing, etc.) put in place?
19. Public health messaging for COVID-19 includes campaigns, posters, and ads that might include messages such as: stay home as much as possible, watch your distance, wear your mask, wash your hands often. Do you think this messaging has been effective in educating you and your friends/peers about risks and prevention associated with COVID-19?
20. What types of services have you accessed during COVID-19 (e.g., shelters, healthcare, social services, mental health)?

<u>If there is enough time in the hour, please include these questions:</u>

21. Describe your educational background? Highest level of education?
22. Have you experienced homophobia, transphobia or biphobia in any way (online, at services, housing, healthcare, family, friends, public) since the COVID-19 pandemic (since March 1, 2020)? Please describe.
23. Describe your main needs during the pandemic? Have you been able to get those needs met?
24. What is helping you get by/make it through/cope with the stress/challenges brought on by the pandemic?
